# Found among Many Places and Sources

**Published:** 1895-12

**Authors:** 


### FOUND AMONG MANY PLACES AND SOURCES.

Secretary Morton has reduced the cost of inspection of animals from one and three-quarter cents per head in 1894 to one and one-tenth cents each during the year 1895. 1,361,800 head of cattle and sheep were inspected this past year. The increase in the exportations of sheep over the year 1894 amounted to over 300 per cent. 5,631,913 more animals were inspected this year over that of 1894.

Molansky, a baker of New York City, bids fair to become famous in the annals of medical literature. His features and extremities are gradually changing in a retrograde manner, resembling those of a monkey, designated in scientific literature as acromegalia. His case has awakened a very great interest among physicians and scientists, and will be watched with increased interest.

In 1896 Mexico will adopt the metric system of weights and measures.

Philadelphia's strength as a medical centre grows with each year, and keeps pace well with her advanced courses of instruction. Some twenty-six hundred students are registered this year in her several medical schools—a gain of nearly three hundred over that of 1894 and 1895.

The very wide difference of opinion among expert veterinarians as to the soundness of a number of the horses at the recent horse-show held in New York City, added an additional interest to the occasion.

An Indiana calf, two months old, has hoofs like a horse.

The Keystone Veterinary Medical Association of Philadelphia and vicinity has adopted an application-blank for its future members to conform to.

A lady in Washington is the possessor of a pug-dog which has been taught to articulate the word "Mum-a."

Another equally interesting psychological fact pertains to a little black-and-tan dog, in the same city, which in running after other dogs whistles somewhat like a naphtha-launch. The upper lip seems to vibrate, but the approximation of the teeth has something to do with its production. The whistle terminates in a short yelp.

